# The macrophage opera: from metabolic prolog to oncogenic crescendo in metabolic dysfunction-associated steatotic liver disease

**DOI:** 10.3389/fmed.2026.1751290

**Published:** 2026-02-18

**Authors:** Gaoling Huang, Dan Li

**Affiliations:** 1 Clinical Medicine, School of Basic Medical Sciences, Naval Medical University, Shanghai, China; 2 Navy Special Medical Center, Naval Medical University, Shanghai, China

**Keywords:** hepatocellular carcinoma, immunometabolism, macrophages, metabolic dysfunction-associated steatohepatitis, metabolic dysfunction-associated steatotic liver disease, polarization, therapeutic targets, tumor-associated macrophages

## Abstract

Metabolic dysfunction-associated steatotic liver disease (MASLD) and its progressive inflammatory subtype, metabolic dysfunction–associated steatohepatitis (MASH), have emerged as a pressing global health crisis, imposing escalating clinical and economic burdens worldwide. The disease continuum progresses from isolated hepatic steatosis through inflammatory steatohepatitis and progressive fibrosis to cirrhosis, markedly elevating the risk of hepatocellular carcinoma (HCC). As key orchestrators of innate immunity and tissue homeostasis, hepatic macrophages dynamically mediate disease initiation, progression, and malignant transformation across this spectrum. This review systematically synthesizes current knowledge on macrophage-centric pathophysiological mechanisms spanning the MASLD–MASH–HCC axis and evaluates state-of-the-art macrophage-targeted therapeutic strategies. We first delineate the developmental origins, functional heterogeneity, polarization plasticity, and spatially resolved architecture of hepatic macrophage populations, emphasizing their transcriptional and phenotypic diversity that extends beyond the traditional M1/M2 paradigm. Stage-specific mechanisms are further elucidated, ranging from lipotoxic stress-induced inflammatory signaling and Kupffer cells(KCs)-dependent monocyte recruitment to crown-like structure formation and the role of disease-associated macrophage subsets. These macrophage-driven processes collectively promote an immunosuppressive niche and confer therapy resistance in established HCC. Finally, emerging therapeutic paradigms aimed at modulating macrophage recruitment, polarization, metabolism, and effector functions via nanomedicine and cell-based modalities are critically appraised. Unraveling the spatiotemporal dynamics of macrophage behavior during MASLD–HCC progression is pivotal for designing stage-specific interventions to halt disease advancement and improve long-term patient survival.

## 1 Introduction

Metabolic dysfunction-associated steatotic liver disease (MASLD, formerly known as non-alcoholic fatty liver disease/NAFLD) and its more severe inflammatory subtype, metabolic dysfunction-associated steatohepatitis (MASH), have emerged as the most prevalent chronic liver diseases (CLDs) worldwide and a critical public health challenge (1). According to the 2021 Global Burden of Disease study, approximately 1.27 billion individuals are affected by MASLD globally, including 510 million who also have type 2 diabetes (2). Recent large-scale meta-analyses indicate that the overall global prevalence of NAFLD/MASLD is approximately 30%. Importantly, pooled estimates demonstrate a marked temporal increase, with prevalence rising from ∼25% in 1990–2006 to as high as 38.0% during 2016–2019 in recent cohorts. This increasing burden is particularly pronounced in low- to middle-sociodemographic index regions (3–5). The pathogenesis is closely linked to persistent energy surplus, obesity, insulin resistance (IR), and related metabolic dysregulation (6).

The spectrum of MASLD ranges from isolated hepatic steatosis (a relatively benign initial stage) to the more advanced steatohepatitis (MASH), which is characterized by hepatocyte injury, inflammation, and fibrosis. The transition to fibrosis and cirrhosis significantly worsens patient prognosis (7). Evidence indicates that the annual incidence of hepatocellular carcinoma (HCC) reaches 2–4% in patients with cirrhosis (3) and remains as high as 0.5–1.5% even among non-cirrhotic MASH patients (8, 9), indicating that the transition from MASH to fibrosis represents a critical turning point in long-term outcomes. Despite the rapidly increasing global burden of MASLD/MASH, effective treatments for moderate to advanced disease remain limited, highlighting the urgent need to elucidate underlying molecular and cellular mechanisms.

Throughout the MASLD-MASH-fibrosis-HCC disease continuum, hepatic macrophages serve as central components of the innate immune system, exerting dynamic and multidimensional regulatory functions that are crucial for disease progression (10). The hepatic macrophage population consists primarily of two major subsets: embryonically derived, self-renewing resident Kupffer cells (KCs) (11) and monocyte-derived macrophages (MoMFs) (12) recruited from the circulation during tissue injury and inflammatory states. These cells exhibit remarkable heterogeneity and plasticity in their developmental origins, surface markers, transcriptional profiles, spatial distribution, and functional states. Through polarization shifts and microenvironmental responses, they participate in critical pathological processes across all disease stages: regulating lipid homeostasis and immune balance during steatotic phases; amplifying inflammation in MASH through KCs activation, monocyte recruitment, crown-like structure formation, and interactions with various immune and non-immune cells; modulating hepatic stellate cell (HSC) activation, proliferation, and extracellular matrix (ECM) deposition during fibrosis; and remodeling the immunosuppressive tumor microenvironment (TME) in HCC, thereby promoting tumor angiogenesis, invasion, metastasis, and influencing therapeutic sensitivity.

Given the central role of macrophages throughout MASLD-HCC progression, targeting these cells has become a promising therapeutic strategy. In recent years, significant advances have been made in interventions targeting macrophage recruitment, polarization, metabolic reprograming, and innovative approaches based on nanotechnology or cell therapy across all disease stages, from steatosis/MASH to fibrosis/cirrhosis and HCC. This review systematically elaborates the key mechanistic roles of hepatic macrophages throughout the MASLD-HCC disease spectrum, analyzes their spatiotemporal dynamic characteristics and functional attributes, with particular focus on their central pathological contributions at each disease stage, and summarizes recent advances in macrophage-targeted therapies. This comprehensive analysis aims to provide a theoretical foundation and translational direction for developing stage-specific, macrophage-centered intervention strategies, ultimately improving long-term clinical outcomes for patients with MASLD-HCC.

## 2 Overview of the origin, polarization, and distribution of macrophages in the liver

### 2.1 Classification of macrophages in the liver: resident KCs, MoMFs, peritoneal macrophages

**FIGURE 1:**
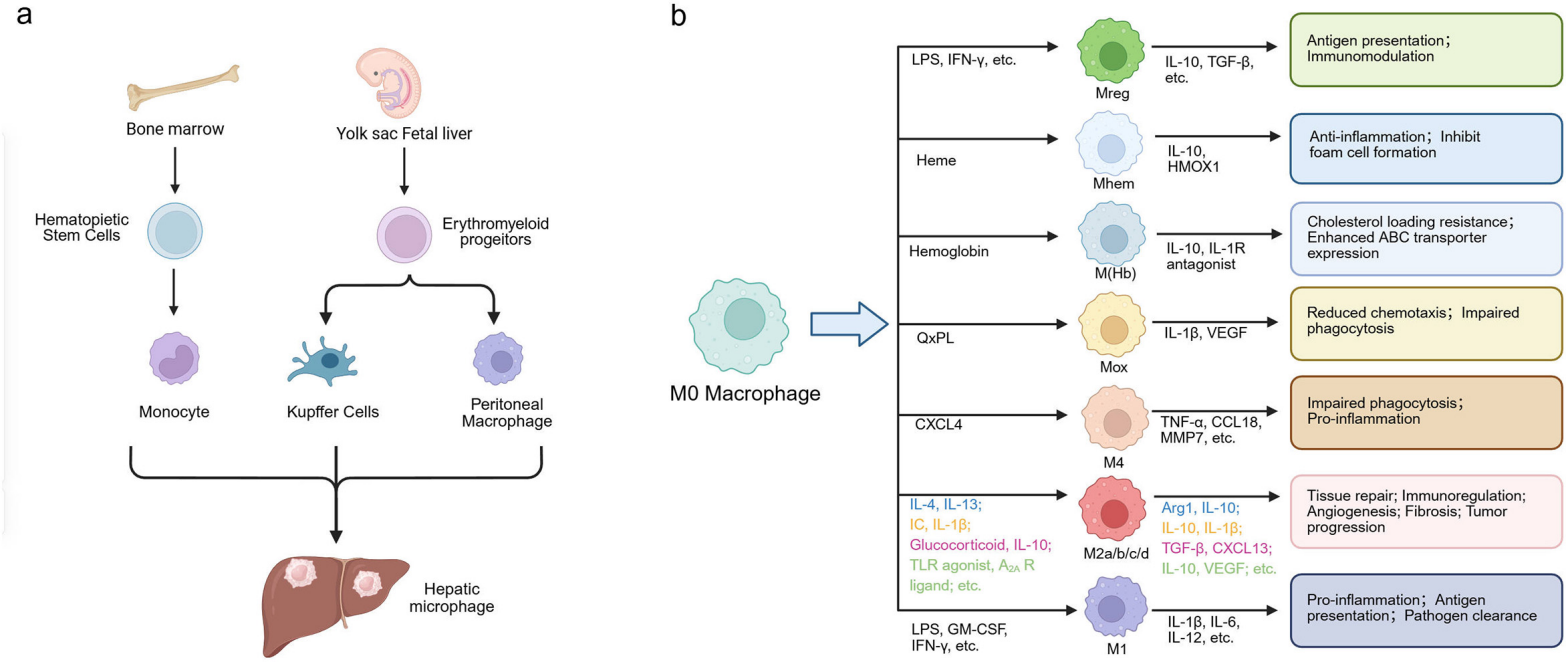
Origin and polarization spectrum of hepatic macrophages. **(a)** Schematic representation of the developmental origins of hepatic macrophages. Liver macrophages arise from two main sources: embryonic yolk sac–derived EMPs, which give rise to resident KCs and peritoneal macrophages, and bone marrow–derived hematopoietic stem cells, which produce circulating monocytes that differentiate into MoMFs during inflammation or injury. **(b)** Polarization spectrum and functional heterogeneity of macrophages. Resting macrophages (M0) exhibit high plasticity and can polarize into various subsets in response to microenvironmental cues. Classical M1 macrophages are pro-inflammatory, whereas alternative M2 macrophages mediate anti-inflammatory and tissue-repair functions. Beyond the M1/M2 paradigm, non-classical phenotypes such as M4, Mox, Mhem, M(Hb), and Mreg display specialized functions in oxidative stress regulation, heme metabolism, and immune modulation. HSCs, hematopoietic stem cells; EMPs, erythromyeloid progenitors; KCs, Kupffer cells; MoMFs, monocyte-derived macrophages; M0, resting macrophage; M1, classically activated macrophage; M2, alternatively activated macrophage; M4, CXCL4-induced macrophage; Mox, oxidized phospholipid–induced macrophage; Mhem, heme-induced macrophage; M(Hb), hemoglobin-induced macrophage; Mreg, regulatory macrophage; IL, interleukin; TNF-α, tumor necrosis factor alpha; TGF-β, transforming growth factor beta; VEGF, vascular endothelial growth factor; GM-CSF, granulocyte-macrophage colony-stimulating factor; IFN-γ, interferon gamma.

Liver macrophages are the core functional units of the liver immune system, characterized by diverse origins and complex phenotypic heterogeneity. They are key cell populations that maintain liver immune homeostasis and regulate pathological processes. Based on their origin and functional characteristics, they are mainly classified into three types: resident KCs, MoMFs, and peritoneal macrophages (13), each playing differentiated roles in liver physiology and pathology (Figure 1a).

KCs are the primary effector cells of the liver’s innate immune defense, originating from embryonic yolk sac erythromyeloid progenitors (EMPs). After maturation, they can self-renew through local proliferation without relying on the replenishment of bone marrow monocytes (10, 14). Functionally, they can sense pathogen invasion and cellular damage signals through pattern recognition receptors (PRRs) such as Toll-like receptors (TLRs), regulating local inflammatory responses; they can also secrete chemokines such as C-C motif chemokine ligand 2 (CCL2) and C-X-C motif chemokine ligand (CXCL10) after liver injury, recruiting peripheral monocytes and inducing their differentiation into mature macrophages, forming a collaborative network that participates in damage repair, inflammation resolution, and immune clearance (14–16).

MoMFs primarily originate from monocytes differentiated from hematopoietic stem cells in the bone marrow, with the spleen also providing supplementation under specific pathological conditions (14, 17). During liver inflammation or injury, monocytes from the bone marrow and spleen are mobilized to the injury site by inflammatory factors such as tumor necrosis factor-alpha (TNF-α), interleukin-6 (IL-6), and CCL2/C-C chemokine receptor 2 (CCR2) chemotactic signals, and differentiate into functionally heterogeneous MoMFs under the regulation of transforming growth factor-β (TGF-β), IL-10, and others (18). Based on the relative expression levels of CD14 and CD16 on the cell surface, human circulating monocytes are commonly classified into three subsets: classical (CD14^ + +^CD16^–^), intermediate (CD14^ + +^CD16^–^), and non-classical (CD14^+/–^CD16^ + +^) (19, 20). The CD14^+^CD16^–^ subtype expresses high levels of TLR4 and NF-κB-related molecules (21), can secrete IL-1β and IL-12 to amplify inflammation, participate in antigen presentation, and regulate pathological processes such as MASH and viral hepatitis. Each subtype can also influence the occurrence, development, and reversal of liver fibrosis by regulating the balance of matrix metalloproteinases (MMP-2, MMP-9) and tissue inhibitors of metalloproteinases (TIMPs) (22).

Abdominal cavity macrophages are located in the subcapsular region of the liver and perihepatic adipose tissue, expressing common macrophage markers such as F4/80, CD64, and CD11b, but not expressing KCs-specific molecules, which is a core distinguishing feature between the two (23). During the liver injury repair phase, they can sense chemokines such as CXCL13 and migrate to the injury site, anchoring through interactions with endothelial cell ICAM-1 and VCAM-1. On one hand, they secrete TGF-β1 to promote the activation and proliferation of HSCs, and on the other hand, they release IL-10 to inhibit excessive inflammation, balancing liver tissue regeneration and injury repair (23).

### 2.2 Polarization characteristics and technical research progress of liver macrophages

The functional phenotype of liver macrophages is dynamically regulated by local microenvironmental signals, and the plasticity of their polarization state is central to maintaining liver immune homeostasis. While the traditional classification into pro-inflammatory M1 and anti-inflammatory M2 types provides a basic framework, it fails to comprehensively reflect the heterogeneity within the complex liver microenvironment (24–26). To capture this complexity, the activation spectrum has been expanded to include M2 sub-lineages (M2a–d) and atypical phenotypes (e.g., M4, Mox, M(Hb), Mhem, Mreg) driven by specific pathological stimuli. We provide a comprehensive summary of the specific markers, cytokine profiles, and functional roles of these diverse subtypes in Table 1 and Figure 1b.

**TABLE 1:** Specific markers, cytokines, and functional roles of macrophage polarization subtypes.

Polarization type	Stimulant	Specific markers	Cytokines	Effect	Reference
M1	LPS, GM-CSF, IFNG, IFN-γ, PAMPs, DAMPs, TNF	CD68, CD80, CD86, CD16/32, MHCII, iNOS	IL-1β, IL-6, IL-12, IL-23, TNF-α, CXCL1∼3, CXCL8∼10, CCL2∼5, CCL11	Performing essential roles in antigen presentation, Th1 initiation, and pathogen clearance, but risking intensified inflammation and tissue damage upon activation.	(220)
M2a	IL-4, IL-13	CD206, MHCII, IL-1R	Arg1, IL-10, TGF-β, CCL17, CCL22	Promoting key processes including cell growth, tissue repair, and endocytosis; additionally driving anti-inflammatory responses, wound healing, Th2 immunity, anaphylaxis, and fibrosis.	(36)
M2b	Immune complex, IL-1β, LPS	CD206, MHCII, CD86	IL-10, IL-1β, IL-6, TNF-α, IL-12^low^	By modulating immune responses, it exerts critical immunoregulatory effects that can paradoxically favor tumor progression and enhance susceptibility to infections.	(221)
M2c	Glucocorticoid, IL-10, TGF-β,	CD206, CD163, TLR1, TLR8	TGF-β, CXCL13, CCL16, CCL18, IL-10, Arg-1,	Through the phagocytic clearance of apoptotic cells and the promotion of fibrosis, it facilitates immunosuppressive activity and executes complex tissue remodeling, a process vital to both repair and pathology.	(222)
M2d	TLR agonist, A_2A_ R ligand	CD206	IL-10, VEGF, IL-12^low^, TNF-α^low^	It promotes tumor progression by facilitating angiogenesis.	(33)
M4	CXCL4	MMP7^+^S100A8^+^, CD206, CD163^–/–^	TNF-α, CCL18, MMP7	It demonstrates impaired phagocytic function coupled with a pro-inflammatory response.	(34)
Mox	QxPL	HO-1, Srxn1, Gclc, Gclm, Txnrd1, Nurr1, Trib3, COX-2	IL-1β, VEGF	Demonstrating reduced efficacy in both chemotactic responses and phagocytic processes.	(223)
M(Hb)	Hemoglobin	CD206, CD163	IL-10, IL-1R antagonist	Exhibiting cholesterol loading resistance and enhanced ATP-binding cassette transporter expression.	(224)
Mhem	Heme	CD163, HMOX1	IL-10, HMOX1	Anti-inflammatory function and inhibition of foam cell formation.	(225)
Mreg	LPS, IFN-γ, immune complex	CD80, MHC-II, DHRS9	IL-10, TGF-β, NOS, IDO	Immune functions encompassing antigen presentation and immunomodulation.	(226)

LPS, Lipopolysaccharide; GM-CSF, Granulocyte-Macrophage Colony-Stimulating Factor; IFNG, Interferon Gamma; IFN-γ, Interferon Gamma; PAMPs, Pathogen-Associated Molecular Patterns; DAMPs, Damage-Associated Molecular Patterns; TNF, Tumor Necrosis Factor; MHCII, Major Histocompatibility Complex Class II; iNOS, Inducible Nitric Oxide Synthase; IL, Interleukin; CXCL, C-X-C Motif Chemokine Ligand; CCL, C-C Motif Chemokine Ligand; MASH, Metabolic Associated Steatohepatitis; CLS, Cellular Lipid Scaffold; Arg1, Arginase 1; TGF-β, Transforming Growth Factor Beta; Th, T Helper; TLR, Toll-Like Receptor; A2 A R, Adenosine A2 A Receptor; VEGF, Vascular Endothelial Growth Factor; MMP, Matrix Metalloproteinase; HO-1, Heme Oxygenase 1; Srxn1, Sulfiredoxin 1; Gclc, Glutamate-Cysteine Ligase Catalytic Subunit; Gclm, Glutamate-Cysteine Ligase Modifier Subunit; Txnrd1, Thioredoxin Reductase 1; Nur1, Nuclear Receptor Related 1; Trib3, Tribbles Pseudokinase 3; COX-2, Cyclooxygenase 2; ATP, Adenosine Triphosphate; HMOX1, Heme Oxygenase 1; IDO, Indoleamine 2,3-Dioxygenase; DHRS9, Dehydrogenase/reductase 9; CCR, CC Chemokine Receptor.

The activation of M1 macrophages relies on multi-pathway cooperation: lipopolysaccharide (LPS) activates the NF-κB pathway through TLR4 to induce pro-inflammatory gene expression (27), while interferon-γ (IFN-γ) promotes the transcription of iNOS and IL-1β via the Janus kinase 1 (JAK1)/signal transducer and activator of transcription 1 (STAT1) signaling pathway (28), and Notch signaling maintains its phenotypic stability (29). Functionally, it highly expresses CD80, CD86, and MHC class II molecules, and plays a role in pathogen defense and inflammation amplification during acute liver infection and early injury by producing nitric oxide (NO) and secreting IL-1β and TNF-α (30). In contrast, the polarization of M2 macrophages is mainly induced by IL-4 and IL-13, regulated by the JAK2/STAT6 pathway to control the expression of Arg-1 and CD206, while TGF-β can further enhance its phenotype through the Smad2/3 signaling pathway (31, 32); it highly expresses CD163 and CD206, secretes IL-10 and TGF-β to inhibit inflammation and promote tissue repair (30), and can be subdivided into M2a (mediating Th2 immune regulation), M2b (bidirectional inflammation regulation), M2c (tissue remodeling), and M2d (promoting angiogenesis) subtypes, supporting liver homeostasis (33).

However, the traditional M1/M2 binary model has significant limitations. Recent single-cell transcriptomics technologies have revealed that liver macrophages do not strictly follow a binary pattern in pathological states such as chronic inflammation and fibrosis, but instead exhibit atypical polarized states along a continuous spectrum. For example, CXCL4-induced M4 macrophages (CD68^+^MMP7^+^S100A8^+^) can regulate vascular smooth muscle cell proliferation and participate in liver vascular lesions (34, 35). There are also Mox types with antioxidant damage and Mhem and M (Hb) subgroups that regulate heme metabolism in atherosclerotic plaques, all of which demonstrate the high plasticity of their polarization being highly regulated by extracellular signals (30, 36). Of particular note, regulatory macrophages (Mregs) represent a functionally distinct macrophage subtype defined by specific molecular characteristics: expression of dehydrogenase/reductase 9 (DHRS9) serves as their specific marker, coupled with high levels of MHC-II and CD80 (37). This phenotypic profile enables their dual capacity for antigen presentation and immunosuppression. They can directly inhibit T-cell proliferation as well as indirectly modulate immune responses through the secretion of immunoregulatory molecules such as IL-10 and TGF-β. Consequently, Mregs play an indispensable role in tissue repair and the maintenance of immune homeostasis (38).

With the development of spatial omics and single-cell transcriptomics technologies, research on liver macrophages has broken traditional limitations (39): studies on human liver based on the 10x Genomics Visium platform show that KCs (CD68^+^MARCO^+^) are enriched in the portal area, while MoMFs (CD68^+^MARCO^+^) are concentrated in the central vein area, with distribution differences closely related to local microenvironment signals (40); research on C57BL/6 mice has also for the first time discovered portal area macrophages (CX3CR1^+^CD63^+^), which play a key role in maintaining immune homeostasis in the portal area and in neuro-immune regulation (41).

At the same time, using Cre-LoxP lineage tracing and Tamoxifen-induced fluorescent labeling technology, researchers clarified the origin and fate of liver macrophages (11): approximately 85% of KCs in the livers of adult mice originate from embryonic EMPs, maintaining their numbers through local self-proliferation (17); MoMFs originate from bone marrow hematopoietic stem cells and migrate into the liver via the bloodstream for differentiation (42). In liver injury repair, these two types of cells participate in microenvironment remodeling and immune balance through signaling interactions, laying a theoretical foundation for understanding their dynamic regulatory mechanisms in liver fibrosis and liver cancer.

## 3 From simple hepatic steatosis to liver cancer: the core role and mechanism of hepatic macrophages at various stages of disease

**FIGURE 2:**
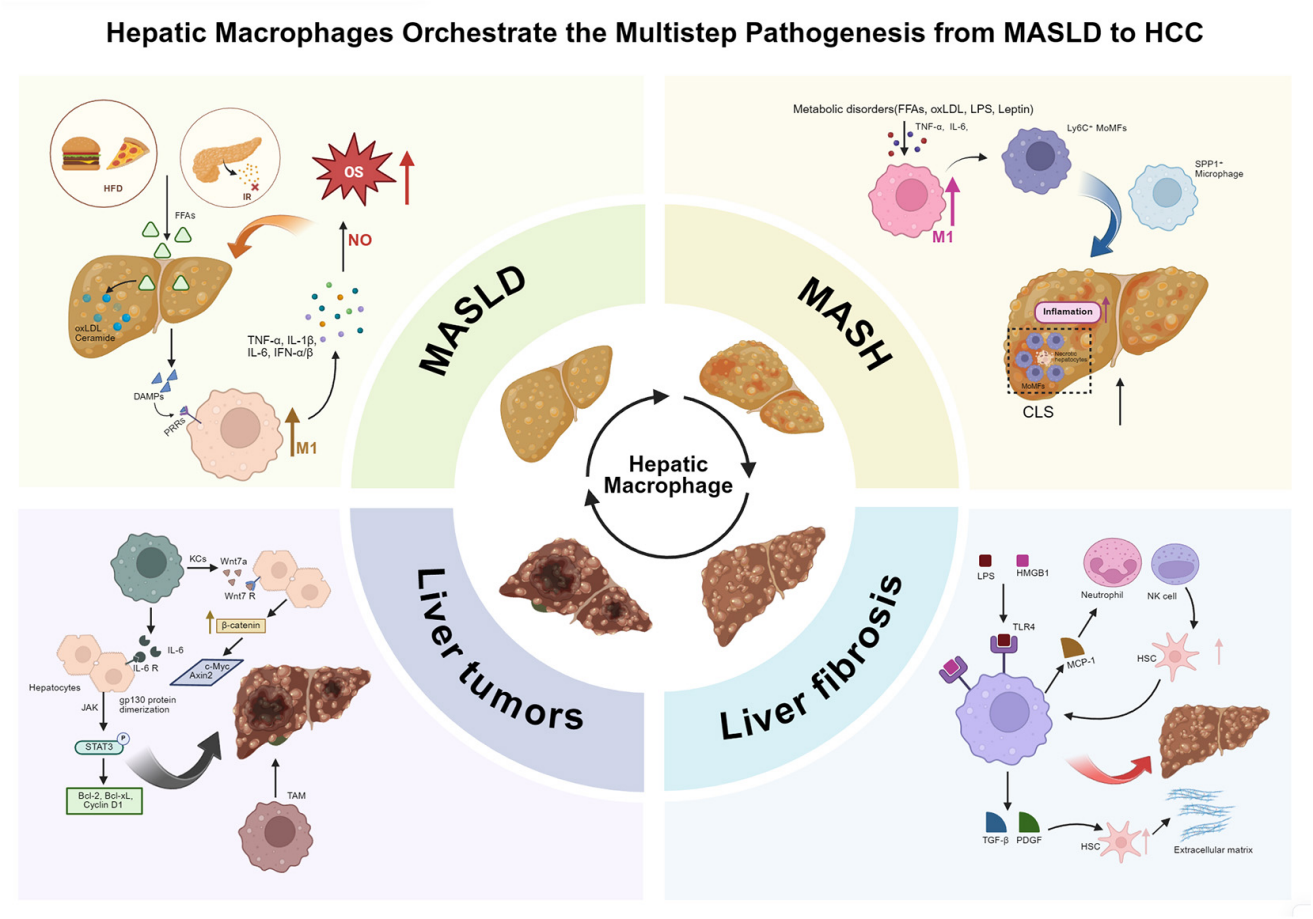
Hepatic macrophages orchestrate the multistage progression from MASLD to hepatocellular carcinoma. Hepatic macrophages dynamically regulate the progression of MASLD through multiple pathological stages, including MASH, liver fibrosis, and liver tumors. (1) In the early stage of MASLD, excessive lipid accumulation caused by insulin resistance and high-fat diet leads to lipotoxic injury, mitochondrial dysfunction, and the release of DAMPs, activating KCs via TLR4/MyD88/NF-κB signaling and promoting the secretion of pro-inflammatory cytokines such as TNF-α, IL-1β, and IL-6. (2) During the transition to MASH, lipid peroxidation products and metabolic stress further promote the polarization of hepatic macrophages toward the M1 phenotype, while the recruitment of Ly6C^+^ MoMFs through the CCR2/CCR5 axis amplifies inflammation and promotes the formation of CLS. These structures act as spatial carriers for chronic inflammation and fibrosis initiation. (3) In liver fibrosis, macrophages activate HSCs through TGF-β, PDGF, and MCP-1 signaling, promoting ECM deposition. Pro-fibrotic SPP1^+^ and TREM2^+^ macrophages accumulate in fibrotic niches, sustaining HSC activation and collagen production, while Ly6C∧low macrophages promote fibrosis regression via MMP-mediated ECM degradation and IL-10 secretion. (4) In liver tumorigenesis, TAMs derived from KCs and MoMFs promote malignant progression by activating the IL-6/JAK/STAT3 pathway, enhancing β-catenin signaling, and secreting pro-angiogenic and immunosuppressive factors that sustain tumor proliferation, angiogenesis, and immune evasion. Collectively, hepatic macrophages act as pivotal regulators linking metabolic stress, inflammation, fibrosis, and oncogenic transformation in the continuum of chronic liver disease. MASLD, metabolic dysfunction-associated steatotic liver disease; MASH, metabolic dysfunction-associated steatohepatitis; HCC, hepatocellular carcinoma; KCs, Kupffer cells; MoMFs, monocyte-derived macrophages; CLS, crown-like structure; HSC, hepatic stellate cell; TAMs, tumor-associated macrophages; DAMPs, damage-associated molecular patterns; TLR4, Toll-like receptor 4; MyD88, myeloid differentiation primary response 88; NF-κB, nuclear factor kappa-light-chain-enhancer of activated B cells; TNF-α, tumor necrosis factor alpha; IL, interleukin; MCP-1, monocyte chemoattractant protein 1; PDGF, platelet-derived growth factor; TGF-β, transforming growth factor beta; MMP, matrix metalloproteinase; ECM, extracellular matrix; STAT3, signal transducer and activator of transcription 3; JAK, Janus kinase; CCR2/CCR5, C-C chemokine receptor type 2/5; TREM2, triggering receptor expressed on myeloid cells 2; SPP1, secreted phosphoprotein 1.

CLD has become a significant public health challenge globally. According to the World Health Organization, it causes approximately 2 million deaths annually, making it the third leading cause of non-communicable disease-related deaths among individuals aged 15–49. Against the backdrop of the global epidemic of obesity and metabolic syndrome, the incidence of CLD continues to rise, presenting a severe prevention and control situation. From a pathological perspective, as illustrated in Figure 2, CLD typically progresses through a sequential spectrum, beginning with simple steatosis, which can evolve into MASH, then progress to liver fibrosis, and ultimately advance to cirrhosis and HCC (43, 44), with significant differences in the molecular regulatory mechanisms and the state of the hepatic immune microenvironment at different stages. Early simple hepatic steatosis is often in a “benign compensatory” state, and the transition to MASH is a critical turning point for the disease moving from the “benign stage” to the “inflammation-driven progression stage.” This turning point, along with the subsequent pathological deterioration of liver fibrosis and carcinogenesis, is primarily driven by the abnormal activation of the hepatic immune microenvironment. As the core functional unit of the hepatic immune microenvironment, the activation state, polarization phenotype, and functional effects of hepatic macrophages dynamically adjust with the pathological stages of CLD, directly influencing the direction of disease progression. Therefore, systematically analyzing the mechanisms of hepatic macrophages at different pathological stages of CLD is not only key to elucidating the patterns of disease progression but also provides an important entry point for developing targeted intervention strategies for CLD.

### 3.1 The role of macrophages in the early stage of MASLD (simple steatosis)

In the early stage of MASLD, during the phase of simple steatosis, hepatic lipotoxicity is the core initiating factor that triggers the liver’s innate immune response. The activation of liver macrophages, particularly the resident KCs, is a key link that connects lipotoxic damage with inflammatory responses and drives disease progression.

When the body experiences abnormal increases in peripheral fat breakdown due to IR and a high-fat diet, a large amount of free fatty acids (FFAs, primarily palmitic acid) floods into the liver via the portal vein. If the FFAs exceed the mitochondrial β-oxidation capacity of liver cells, they will accumulate with cholesterol in the cytoplasm (45); some unmetabolized lipids will also generate toxic products such as oxidized low-density lipoprotein (oxLDL) and ceramides through lipid peroxidation (46, 47)—these products can damage the mitochondrial membrane potential, induce endoplasmic reticulum stress, and directly harm liver cells (48, 49). They can also activate caspase-1 mediated pyroptosis and caspase-3 mediated apoptosis, prompting damaged liver cells to release damage-associated molecular patterns (DAMPs) such as high mobility group box 1 (HMGB1), heat shock protein 70 (HSP70), and uric acid crystals, providing “danger signals” for immune activation (14, 50). The DAMPs in the liver microenvironment can bind to PRRs on the surface of KCs, initiating their activation: HMGB1 activates the TLR4/MyD88/NF-κB pathway by binding to TLR4 and receptor for advanced glycation end products (RAGE) (51), promoting the nuclear translocation of NF-κB dimers and initiating the transcription of pro-inflammatory factors such as IL-1β, TNF-α, and IL-6; uric acid crystals activate the TLR9/IRF7 pathway, promoting the secretion of type I interferons (52); oxLDL enhances TLR4 signaling through scavenger receptor A1 (SR-A1), ultimately forming a positive cascade of “lipotoxic injury-DAMPs release-KCs activation-pro-inflammatory factor secretion” (53), laying the foundation for the transition from MASLD to MASH.

Activated KCs amplify liver inflammation through a dual effect: on one hand, they continuously secrete TNF-α and IL-1β via the NF-κB and MAPK/ERK pathways, which directly bind to liver cell receptors to induce apoptosis and regulate the activation of HSCs through paracrine signaling (47, 54); on the other hand, under the stimulation of lipotoxic products, they polarize toward the M1 pro-inflammatory type, upregulating CD80, CD86, and inducible nitric oxide synthase (iNOS), while downregulating CD206 and arginase-1 (Arg-1) (55, 56), which enhances antigen presentation and pro-inflammatory capacity, and exacerbates oxidative stress through excessive production of NO by iNOS, forming a positive feedback loop of “oxidative damage-inflammation activation,” accelerating hepatocyte necrosis and the initiation of fibrosis. Furthermore, the cytokines secreted by KCs also regulate the recruitment of peripheral immune cells: TNF-α upregulates ICAM-1 and VCAM-1 in liver endothelial cells, enhancing monocyte adhesion (57); IL-1β, IL-6, and IL-18 activate monocyte CXCR2 and CCR2, guiding their migration to the injury site (18). The infiltrating monocytes differentiate into MoMFs under the regulation of local TGF-β, IL-10, and FFAs (12, 58)—these cells express high levels of TLR4 and SR-A1, respond more strongly to lipotoxic signals (12), and can amplify inflammation by secreting IL-6 and CCL2, releasing MMPs and tissue cathepsins to participate in ECM remodeling (59), ultimately driving the progression of MASLD from simple steatosis to MASH, becoming a key pathological node in its progression to liver fibrosis and cirrhosis.

### 3.2 The role of liver macrophages in the MASH stage: the dominators of inflammation and immune activation

#### 3.2.1 Connecting liver inflammation and metabolic disorders: the core link regulating IR

MASH is a critical progression stage of MASLD, characterized by hepatocellular steatosis, ballooning degeneration, lobular inflammation, and progressive fibrosis. The core of its development is the vicious cycle of “lipotoxicity—immune activation—metabolic disorder,” with liver macrophages (resident KCs and MoMFs) serving as the central hub connecting metabolic stress and inflammation.

In the interaction between inflammation and metabolic disorders, the abnormal activation of KCs is key to the exacerbation of IR in the MASH stage, while the worsening of IR drives the transformation of MASLD into MASH (60). In the MASH stage, lipotoxic substances such as FFAs and oxLDL accumulate in the liver and signal through the leptin receptor (LEPR) on the surface of KCs, prompting KCs to release TNF-α and IL-6 (61). These factors not only promote the polarization of KCs toward the M1 type but also interfere with insulin signaling through two pathways: first, by inducing serine phosphorylation of insulin receptor substrate (IRS), which reduces insulin sensitivity in hepatocytes (62, 63); second, by inducing mitochondrial dysfunction and endoplasmic reticulum stress, which block downstream signaling (64). Clinical evidence shows that serum levels of TNF-α and IL-6 in MASH patients are significantly positively correlated with the degree of IR (*r* = 0.58–0.65). Additionally, the gut-liver axis plays a bridging role: in MASH patients, gut microbiota dysbiosis and impaired intestinal barrier allow bacterial endotoxins (LPS) to enter the liver via the portal vein, activating the TLR4/MyD88/NF-κB pathway through TLR4 on the surface of KCs (65), which exacerbates local hepatic IR and weakens insulin sensitivity in peripheral tissues, forming a cross-organ metabolic inflammatory regulatory network.

#### 3.2.2 Activation of KCs and recruitment of monocytes: the dual mechanism of MASH inflammation initiation

In the initiation phase of MASH inflammation, the activation of KCs is driven by a synergy of endogenous lipotoxic signals and exogenous gut-derived signals. On one hand, DAMPs (such as HMGB1, HSP70) and unmetabolized lipids released from hepatocyte lipotoxic injury, in conjunction with the PRRs on the surface of KCs (TLR4, SR-A1), initiate immune activation (10); on the other hand, gut-derived LPS serves as a key amplifier of KC activation (66)—*in vitro* experiments show that co-treatment of triglycerides with LPS results in a higher expression of pro-inflammatory factors in KCs than LPS stimulation alone (67); *in vivo* studies indicate that KCs from mice fed a choline-deficient amino acid-sufficient (CDAA) diet exhibit stronger secretion of pro-inflammatory factors and lymphocyte recruitment capabilities under LPS stimulation (68).

The sensitivity of KCs to LPS increases during MASH, which is associated with the activation of X-box binding protein 1 (XBP1) mediated by reactive oxygen species (ROS) (69): in primary rat macrophages, LPS induces inflammation through the ROS-XBP1 pathway; in the livers of MASH mice fed a high-fat high-cholesterol diet, the accumulation of ROS rises in tandem with the activation of XBP1 (70). The LPS-TLR4 pathway is central to the interaction of the gut-liver axis (65), with an increase in TLR4^+^KCs in the livers of MASH patients, and the expression of TLR4 in KCs of mice fed a methionine-choline deficient diet increases fivefold (66, 71); specific depletion of KCs can inhibit the high expression of TLR4 and alleviate liver lesions (66).

As the disease progresses, KCs are depleted due to persistent lipotoxicity and inflammatory apoptosis, and the body recruits peripheral blood Ly6C^+^ monocytes into the liver through the CCR2/CCR5 chemotactic axis, where they differentiate into MoMFs (72). MoMFs highly express TLR4 and SR-A1, responding more strongly to lipotoxicity and LPS, secreting pro-inflammatory factors that amplify inflammation, and activating HSCs through the TGF-β/Smad and MerTK pathways (73). This inflammatory relay of “KCs depletion → MoMFs recruitment” is a critical node in the transformation of MASH from “inflammation—fibrosis.”

#### 3.2.3 Crown-like structures and the synergy of multiple immune cells: the spatial carrier of chronic inflammation in MASH

When KCs are depleted and MoMFs are extensively infiltrated, liver tissue forms MASH-specific CLS—spherical structures where MoMFs aggregate around necrotic/steatotic hepatocytes (74, 75). The number of these structures is positively correlated with ballooning degeneration of hepatocytes, lobular inflammation, and the degree of fibrosis, making them a hallmark pathological feature of MASH (76). CLS serves both damage clearance and inflammation amplification functions: on one hand, MoMFs phagocytize cell debris and lipid droplets through SR-A1 and CD36, with triggering receptor expressed on myeloid cells 2^+^ (TREM2^+^) macrophages playing a dominant role due to their high expression of phagocytosis-related molecules (77); on the other hand, CLS acts as a “hotspot” for pro-inflammatory factors, enhancing inflammatory signals through spatial aggregation and recruiting more immune cells.

At the same time, CLS mediates the cooperation of multiple immune cells: MoMFs secrete CXCL1 and release extracellular vesicles containing mtDNA, guiding and activating neutrophils (78, 79), which exacerbate hepatocyte damage through ROS and neutrophil extracellular traps (80); MoMFs also activate CD4^+^/CD8^+^ T cells to trigger cytotoxic responses through antigen presentation. In summary, the cellular lipid scaffold (CLS) transforms acute inflammation into chronic inflammation through the dual mechanisms of “structural fixation and cellular cooperation,” and serves as a key mediator linking lipotoxic damage to the progression of metabolic associated steatohepatitis (MASH).

#### 3.2.4 New subtypes of macrophages: specific regulators of MASH pathological progression

In the chronic inflammatory microenvironment of MASH, the phenotypes and functions of macrophages are highly heterogeneous, making the traditional M1/M2 classification inadequate. Single-cell transcriptomics has facilitated the discovery of pathology-specific subtypes. Among them, TREM2^+^ macrophages (lipid-associated macrophages) are enriched in CLS and fibrotic foci (81), with a significant increase in number in both human and mouse MASH models, exhibiting both lipid droplet phagocytosis and inflammatory regulation—deletion of TREM2^+^ macrophages exacerbates liver fibrosis, while overexpression alleviates the pathological process (82).

Another subtype of secreted phosphoprotein 1^+^ (SPP1^+^) macrophages has a clear pro-fibrotic function: located in the high-grade fibrotic areas of MASH liver tissue, they highly express SPP1 and CD9, activating pro-fibrotic signaling pathways by binding to surface receptors on HSCs, promoting collagen deposition and accelerating fibrosis. This type of TREM2^+^/SPP1^+^ co-expressing pro-fibrotic macrophages is an active participant in the progression of MASH and has become a potential therapeutic target and biomarker (83–85). The discovery of this novel subtype expands the understanding of the immune mechanisms in MASH and provides new directions for targeted interventions.

### 3.3 The role of macrophages in the stage of liver fibrosis

#### 3.3.1 The pro-fibrotic role of macrophages in liver fibrosis

As core regulators in the process of liver fibrosis, macrophages play a pivotal role throughout the disease via multidimensional mechanisms, forming a cascade response of “perceiving damage—activating HSC—regulating metabolism—maintaining inflammation.” In the initiation phase of fibrosis, macrophages activate HSCs through dual pathways: in the exogenous pathway, gut-derived LPS activates both KCs and HSC via TLR4 (86, 87); in the endogenous pathway, DAMPs such as HMGB1 released by hepatocytes synergistically induce macrophages to secrete TGF-β and platelet-derived growth factor (PDGF) (14), with the former promoting collagen synthesis and the latter driving HSC proliferation and regulating the MMP/TIMP balance (88). Meanwhile, macrophages recruit immune cells by secreting chemokines such as monocyte chemoattractant protein 1 (MCP-1) and TNF-α, forming an initial cycle of “damage—immunity—HSC activation.”

As the disease progresses, macrophages drive the worsening of fibrosis through a refined division of subgroups: in terms of ECM metabolism, they promote collagen synthesis via the TGF-β/Smad pathway, enhance HSC proliferation through the PDGF/PI3K/Akt pathway (87), while secreting TIMP-1 and inhibiting MMP activity, disrupting the ECM metabolic balance (89); in terms of inflammation maintenance, activated HSCs attract macrophages through the CCL2/CCL5 chemokines, and the ROS produced by these macrophages further activate HSCs and exacerbate hepatocyte damage, forming a vicious cycle (90, 91). Among MoMFs, the Ly6C^hi^ subtype aggregates through the CCL2/CCR2 axis and releases ROS (92), iNOS, and its metabolic product NO to directly activate HSCs (93), while the CD14^+^CD16^–^ subtype promotes the process by continuously releasing inflammatory factors (22). In addition, macrophages also promote Th17 differentiation through IL-6 and induce Th2 responses via IL-4, further strengthening HSC activation through Th17/Treg imbalance (22). This finely tuned regulatory network dominated by macrophages not only explains the key mechanisms behind the continuous progression of liver fibrosis but also provides a new theoretical basis for targeted therapy.

#### 3.3.2 The antifibrotic role of macrophages in liver fibrosis

Multiple studies have confirmed that liver fibrosis is reversible, and macrophages are key regulators of its regression (94), primarily functioning through three core mechanisms. In inhibiting the activity of HSCs, macrophages reduce ECM production through dual pathways: first, by secreting anti-inflammatory factors such as IL-10, which directly suppress HSC activation and proliferation to decrease ECM deposition (95); second, by recruiting natural killer (NK) cells to induce apoptosis in activated HSCs while also secreting TNF-related apoptosis-inducing ligand (TRAIL) (96), which can downregulate the expression of TIMPs in HSCs, further enhancing the apoptotic effect on HSCs (97). In promoting ECM degradation, macrophages (especially resident KCs) secrete MMP-2, MMP-9, directly participating in the breakdown of fibrotic scar tissue (98); during the regression phase of fibrosis, a subpopulation of Ly6C low-expressing macrophages significantly accumulates in the mouse liver, characterized by reduced secretion of pro-inflammatory factors and high expression of MMP-9 and MMP-2, further strengthening ECM degradation to accelerate fibrosis regression (99). In regulating immune balance, macrophages modulate T cell subset differentiation through cytokine secretion: they secrete IL-10 to promote the differentiation of regulatory T cells (Tregs) (100), which can directly inhibit HSC activation and proliferation (101), and alleviate chronic liver inflammation by suppressing Ly6C high-expressing pro-inflammatory macrophages (102); they secrete IL-12 to promote the differentiation of helper T cells 1 (Th1) (103), where the IFN-γ produced by Th1 can reduce HSC activation and ECM accumulation, and also enhance NK cell activity, synergistically inducing HSC apoptosis to drive fibrosis regression (96).

### 3.4 The role in the occurrence and development of liver cancer: immune evasion and tumor-promoting microenvironment formation

#### 3.4.1 The role of macrophages in the occurrence stage of liver cancer: inflammation regulation and tumor initiation

Macrophages play a central role in the tumor initiation process during the occurrence stage of liver cancer through a dual mechanism of inflammation-driven and signal regulation. In the context of CLD, the resident KCs in the liver are continuously activated, establishing an inflammation-tumor transformation bridge by releasing a group of pro-inflammatory factors centered around IL-6 (104, 105). IL-6 induces the expression of key genes such as Bcl-2 and Cyclin D1 by activating the JAK/STAT3 signaling pathway (106), promoting hepatocyte proliferation and inhibiting apoptosis, thereby directly driving tumor occurrence (104). Furthermore, in mouse models with monocyte/macrophage-specific IL-6 deficiency, the occurrence of CCl_4_-induced spontaneous liver cancer is significantly suppressed, confirming the indispensable role of macrophage-derived IL-6 in HCC initiation from a genetic perspective (107). In metabolically associated liver cancer, macrophages drive tumor initiation through the Wnt/β-catenin pathway (108). In a lipid accumulation microenvironment, infiltrating macrophages secrete ligands such as Wnt7a, activating hepatocyte β-catenin signaling and promoting the expression of tumor initiation-related genes such as Axin2 and c-Myc (109). Selective depletion of macrophages using liposomal chlorophosphonates can lead to a significant reduction in Wnt signaling and completely inhibit tumor occurrence (110), proving that macrophages are the primary source of Wnt signaling induced by fatty degeneration, thereby elucidating a new mechanism for the increased risk of HCC in obese individuals. Notably, specific molecules exert anti-cancer effects by regulating macrophage function: Leukocyte Cell-Derived Chemotaxin 2 inhibits tumor initiation by limiting the activity of inflammatory monocytes (111); the CCL2-CCR2 axis-mediated monocyte recruitment can promote the clearance of senescent hepatocytes (112); and the interaction between macrophages and CD4 + T cells, as well as the activation of the endocannabinoid system, has also been confirmed to have anti-tumor potential (113, 114). These findings reveal a dual regulatory network of macrophages in the occurrence of liver cancer, providing new targets for liver cancer prevention strategies.

#### 3.4.2 The role of macrophages in the development stages of liver cancer: from microenvironment regulation to multidimensional tumor-promoting effects

**FIGURE 3:**
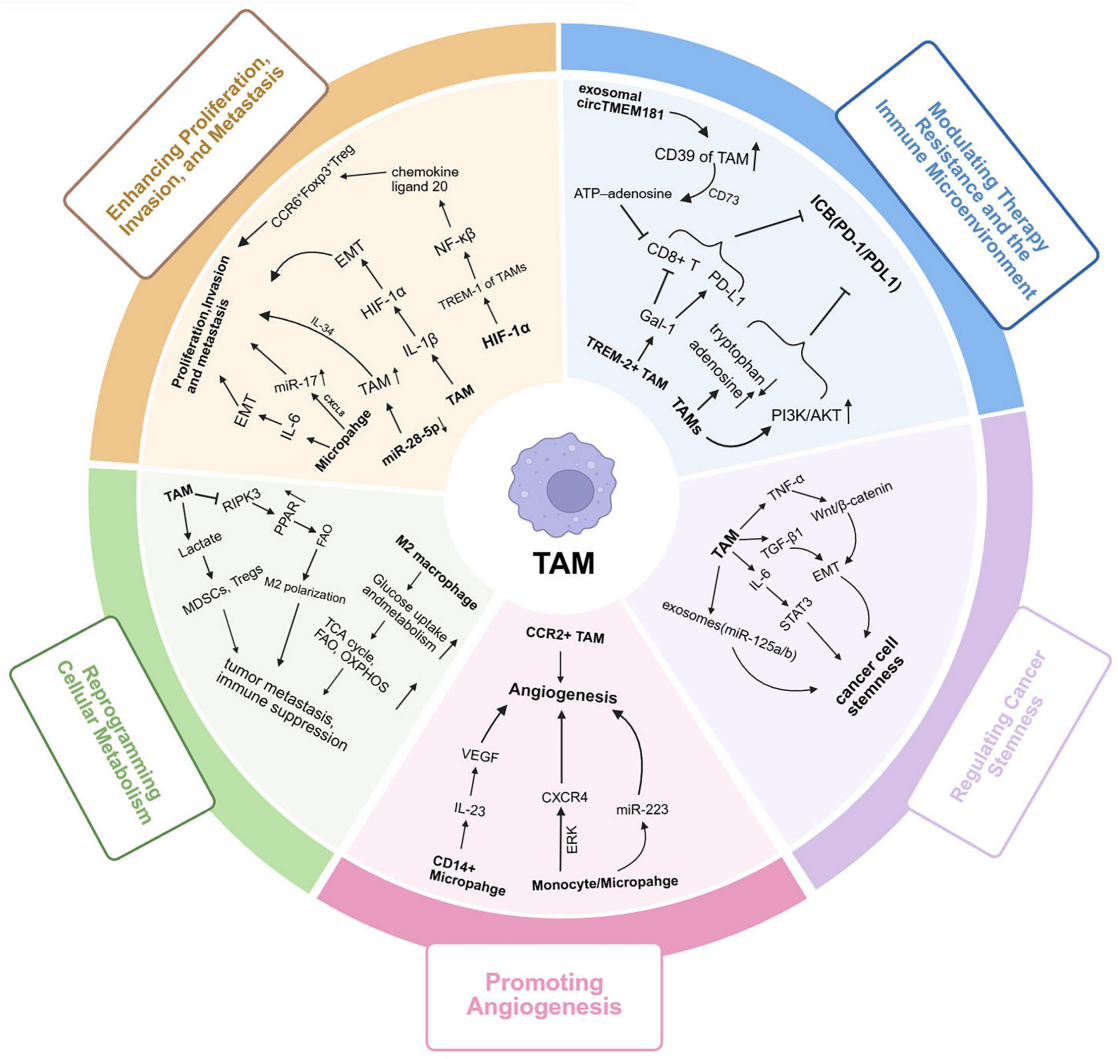
Multifaceted tumor-promoting roles of tumor-associated macrophages (TAMs) in liver cancer progression. TAMs play diverse and dynamic roles in promoting liver cancer progression through multiple interconnected mechanisms: (1) TAMs are predominantly polarized toward the M2-like phenotype under stimulation by IL-4, IL-10, and TGF-β, creating an immunosuppressive and tumor-promoting microenvironment; (2) TAMs secrete IL-6, IL-1β, and TNF-α, activating the NF-κB and HIF-1α signaling pathways, which promote EMT and enhance tumor cell proliferation, invasion, and metastasis; (3) CCR2^+^ TAMs and CD14^+^ macrophages release angiogenic factors such as VEGF, IL-23, and CXCR4, which activate ERK and PI3K/Akt cascades to stimulate endothelial cell proliferation, migration, and neovascularization; (4) TAMs undergo metabolic reprograming characterized by enhanced glycolysis, fatty acid oxidation, and OXPHOS, which provide energy for tumor growth and maintain M2 polarization; (5) Through PD-1/PD-L1, CD39/CD73 adenosine metabolism, and Galectin-1 signaling, TAMs suppress cytotoxic CD8^+^ T cell activity, promote immune evasion, and induce therapy resistance; (6) TAM-derived cytokines (IL-6, TGF-β, TNF-α) and exosomal miRNAs (e.g., miR-125b) activate STAT3 and Wnt/β-catenin pathways, enhancing cancer stemness, self-renewal ability, and drug resistance in liver cancer cells. Collectively, TAMs orchestrate a multidimensional tumor-promoting network integrating inflammation, angiogenesis, metabolism, immune suppression, and stemness regulation, driving the initiation and malignant progression of liver cancer. TAMs, tumor-associated macrophages; IL, interleukin; TGF-β, transforming growth factor beta; TNF-α, tumor necrosis factor alpha; NF-κB, nuclear factor kappa-light-chain-enhancer of activated B cells; HIF-1α, hypoxia-inducible factor 1 alpha; EMT, epithelial–mesenchymal transition; VEGF, vascular endothelial growth factor; CXCR4, C-X-C chemokine receptor type 4; PI3K, phosphoinositide 3-kinase; Akt, protein kinase B; ERK, extracellular signal-regulated kinase; OXPHOS, oxidative phosphorylation; PD-1, programed cell death protein 1; PD-L1, programed death-ligand 1; STAT3, signal transducer and activator of transcription 3; Wnt, Wingless-type MMTV integration site family; miRNA, microRNA.

During the occurrence and development of HCC, tumor-associated macrophages (TAMs) serve as core regulatory components of the TME. Through mechanisms such as phenotypic plasticity, intercellular communication, and metabolic reprograming, they play a key role in various processes including tumor proliferation, invasion and metastasis, angiogenesis, and treatment resistance (Figure 3). The functional state of TAMs is dynamically regulated by a complex signaling network within the TME, driving the malignant progression of liver cancer in a multidimensional and stage-specific manner.

##### 3.4.2.1 Polarization and infiltration of TAMs: tumor-promoting subtype dominated microenvironment remodeling

In the TME of liver cancer, TAMs exhibit high heterogeneity and phenotypic plasticity, with their polarization dynamics and infiltration being central to TME remodeling. Under the regulation of specific signals within the TME (such as IL-4 and IL-13 secreted by Th2 cells, and IL-10 and TGF-β produced by tumor cells), macrophages polarize toward an M2-like phenotype and dominate the TAM population, creating a microenvironment conducive to tumor growth and suppressing immune attacks (115).

This phenotypic transformation is accompanied by dynamic changes in surface markers: during the early infiltration, pro-inflammatory macrophages exhibit a high CD11b, low F4/80, and high Ly6C phenotype (similar to anti-tumor M1 type) (14, 17, 116); as the tumor progresses, they differentiate into strongly pro-tumor M2-like TAMs, with a phenotype shift to low CD11b, high F4/80, and low Ly6C, making Ly6C a key marker for tracking the functional evolution of TAMs (117, 118). Furthermore, the heterogeneity of TAMs is related to their dual origin: resident KCs derived from the embryonic yolk sac provide an initial pro-TME for maintaining liver cancer stemness in the early stages of liver cancer (119); as the tumor progresses, macrophages differentiated from infiltrating bone marrow-derived monocytes become the main supplementary source of the TAM pool. The functions of these two types of macrophages overlap and differ at various stages of the tumor, and their coexistence and interaction constitute a complex functional lineage of macrophages in the TME, profoundly influencing disease progression and treatment response.

##### 3.4.2.2 TAMs orchestrate liver cancer progression by driving proliferation, invasion and metastasis

TAMs promote the proliferation and invasion of HCC through various molecular mechanisms. In terms of proliferation regulation, M2-type TAMs serve as an important source of cytokines, continuously secreting IL-6 to activate the JAK2/STAT3 signaling pathway within tumor cells in a paracrine manner. The sustained activation of this pathway can upregulate the expression of key target genes such as Bcl-2 and Cyclin D1, thereby enhancing the anti-apoptotic capacity of tumor cells and promoting their abnormal proliferation (104, 106). In mediating tumor invasion and metastasis, TAMs effectively degrade ECM components such as type IV collagen by highly expressing MMP-2 and MMP-9, disrupting the integrity of the basement membrane (120). This process not only creates favorable conditions for the local infiltration of tumor cells but also provides a molecular basis for their distant metastasis. Additionally, other proteases and cytokines secreted by TAMs collectively form a microenvironment that promotes tumor invasion, accelerating the malignant progression of liver cancer.

##### 3.4.2.3 The promoting role of TAMs in tumor angiogenesis

TAMs play a key role in the angiogenesis of HCC. A subset of TAMs expressing the Tie-2 receptor promotes the proliferation, migration, and lumen formation of endothelial cells by releasing vascular endothelial growth factor (VEGF) and MMP-12 (121). Preclinical studies have shown that CCR2 + TAMs specifically accumulate in the tumor-normal tissue interface, and the absence of this subset significantly inhibits pathological angiogenesis (122).

Under the stimulation of the tumor-specific hypoxic microenvironment, the stability of hypoxia-inducible factor-1α (HIF-1α) in TAMs is enhanced, leading to nuclear translocation and further promoting the expression of angiogenesis-related genes (123). This process forms a continuously self-reinforcing positive feedback loop, making TAMs core regulatory factors in maintaining tumor angiogenesis. TAMs collaboratively construct a complex network that promotes angiogenesis, providing essential blood supply support for the growth and metastasis of liver cancer.

##### 3.4.2.4 Synergistic metabolic reprograming of TAMs drives tumor malignant tumor progression

The pro-tumor activities of TAMs are tightly coupled to their metabolic plasticity (124). Rather than adopting a fixed metabolic phenotype, TAMs sense and integrate TME cues—including hypoxia, nutrient availability, and cytokine signals—and rewire their metabolic programs accordingly (124, 125). In most settings, TAM metabolism shows a bias toward either glycolysis or mitochondrial oxidative metabolism [fatty acid oxidation (FAO) and oxidative phosphorylation (OXPHOS)], instead of a uniform, simultaneous increase of both pathways (124, 125). In the conventional polarization framework, M1-like and M2-like phenotypes represent two ends of a continuum: M1-like states are typically glycolysis-leaning, whereas M2-like states more often favor FAO/OXPHOS to support immunosuppressive and tissue-repair functions (126–129). Importantly, single tumors frequently exhibit spatially heterogeneous TAM states, and distinct metabolic programs can coexist and interconvert in response to local microenvironmental signals.

Under hypoxia and inflammatory cues (e.g., LPS and IFN-γ), a subset of TAMs shifts toward an M1-like program. HIF-1α stabilization promotes transcriptional induction of Regulated in Development and DNA Damage Responses 1 (REDD1) and glycolytic machinery (e.g., GLUT1 and LDHA), thereby increasing glycolytic flux and supplying rapid energy and biosynthetic intermediates required for activation (130–132). In parallel, mitochondrial oxidative metabolism is often relatively constrained, producing a glycolysis-biased metabolic profile. Consistent with this model, REDD1 inhibition disrupts the glycolytic program and is associated with TAM dysfunction and aberrant tumor angiogenesis (123, 133).

Conversely, in TMEs enriched in immunosuppressive mediators such as IL-10 and TGF-β, TAMs are more likely to adopt a FAO/OXPHOS-leaning program with relatively lower glycolytic activity (127). Lipid handling provides a representative example. TAMs can internalize oxLDL via scavenger receptors; these lipids fuel FAO and, at the same time, promote the expression of immunoregulatory genes such as Arg-1 and IL-10, reinforcing an immunosuppressive TAM state (53, 127, 134). Amino acid–related cues further stabilize this phenotype: tumor-derived adenosine together with TAM-derived granulocyte-macrophage colony-stimulating factor (GM-CSF) can converge on PI3K/AKT signaling, supporting the maintenance and expansion of immunosuppressive TAM states (134, 135).

Taken together, TAM metabolic reprograming is best viewed as a state-linked and context-dependent process. Along a functional continuum, TAMs dynamically tune the balance between glycolysis- and FAO/OXPHOS-leaning programs to sustain survival and immunoregulatory functions within the TME, thereby facilitating tumor progression (125, 127).

##### 3.4.2.5 TAMs regulation of treatment resistance and immune microenvironment

TAMs are key regulators that shape the immunosuppressive TME and mediate resistance to various therapies. Their regulatory mechanisms are primarily reflected in the following three aspects. In the treatment with immune checkpoint inhibitors, TAMs induce resistance through multiple mechanisms. Studies have shown that the aberrant activation of the Wnt/β-catenin signaling pathway in tumor cells is an important factor leading to primary resistance to PD-1/PD-L1 inhibitors (136–138), and TAMs are the main source of Wnt ligands in the TME (109). The sustained β-catenin activation driven by TAMs can lead to T cell exclusion, rendering immune checkpoint inhibitors ineffective (137). This mechanism also explains why such resistant tumors exhibit sensitivity to treatment strategies targeting macrophages, such as CSF1R inhibitors (139).

In shaping the immune microenvironment, TAMs play a protective role by constructing an “immune evasion barrier.” In the early stages of liver cancer, specific tissue-resident macrophages gather around the cancerous lesions, not only secreting pro-tumor factors but also inducing the expansion and activation of regulatory T cells (Treg), thereby forming an inhibitory immune microenvironment locally in the tumor, which weakens the anti-tumor function of cytotoxic T cells (140).

In the field of targeted therapy, TAMs are also involved in resistance mechanisms. In anti-VEGF therapy, the accumulation of TAMs is positively correlated with resistance. When VEGF signaling is blocked, TAMs can rapidly upregulate other pro-angiogenic factors (such as FGF, PGF, etc.) to compensate and maintain tumor vascular survival. The combined use of CSF1R inhibitors or CCR2 inhibitors to block macrophage function can significantly enhance the efficacy of anti-VEGF therapy (140). Similarly, in the treatment with the multi-kinase inhibitor sorafenib, TAMs can weaken drug efficacy by activating alternative survival signaling pathways (such as EGFR, IGF-1R, etc.), while the combination of zoledronic acid or lenvatinib can effectively reverse such resistance (141, 142).

##### 3.4.2.6 TAMs regulate the stemness of liver cancer cells

Liver cancer stem cells (LCSCs) are pivotal drivers of HCC heterogeneity and hierarchical organization, critically contributing to tumor recurrence, metastasis, and treatment resistance. Within the TME, TAMs modulate LCSC properties through a multi-layered regulatory network. Specifically, TAM-derived IL-6 activates STAT3 to promote LCSC expansion, while TGF-β1 induces epithelial–mesenchymal transition to confer stem-like traits (143). Furthermore, TNF-α triggers chromosomal instability in liver progenitor cells via modulation of ubiquitin D and checkpoint kinase 2 (144); in addition, it enhances their self-renewal through the TNFR1/Src/STAT3 axis (145). At the cellular interaction level, tumor-initiating cells actively recruit M2-type TAMs early in tumorigenesis through activation of the Hippo pathway effector Yes-associated protein, establishing a microenvironment conducive to stem cell maintenance (146). Through extracellular vesicle communication, TAM-derived exosomes enhance HCC cell proliferation and stemness by downregulating miR-125a/b, which targets CD90 to suppress stem cell properties (147). Collectively, these mechanisms delineate an integrated and cross-regulatory network through which TAMs orchestrate liver cancer stemness, offering a conceptual framework for understanding HCC progression and informing targeted therapeutic development.

##### 3.4.2.7 TAM-driven dysfunction and exhaustion of CD8^+^ T cells

During the development and progression of MASLD-associated HCC, sustained lipotoxicity, hypoxia, and chronic inflammation collectively drive the TME toward an increasingly immunosuppressive state, in which direct crosstalk between TAMs and cytotoxic CD8^+^ T cells often represents a critical point of antitumor immune failure (148). As tumors progress, TAMs are more likely to acquire immunosuppressive programs and continuously impair CD8^+^ T-cell proliferation, cytotoxic output, and effector persistence (149–155). At the level of cell–cell contact, TAMs constitute an important source of PD-L1 within tumor tissues; persistent activation of PD-1 signaling can promote hallmarks of CD8^+^ T-cell exhaustion, such as reduced effector cytokine secretion and diminished killing capacity. In addition, TAMs can further exacerbate CD8^+^ T-cell dysfunction through other inhibitory receptor–associated pathways, including TIGIT-related axes, thereby establishing a multilayered suppressive network (149, 151, 152). Metabolically, TAMs can deplete arginine by producing arginase-1 (Arg1), which directly constrains CD8^+^ T-cell proliferation and effector differentiation and, under sustained stimulation, promotes a shift toward terminal exhaustion (149, 154). Moreover, TAM-supported hypoxic and acidified microenvironments can cooperate with the CD39–adenosine axis to amplify immunosuppressive adenosine signaling and further dampen T-cell activation and effector function; meanwhile, TAM-derived reactive oxygen species (ROS) can induce mitochondrial stress and metabolic disturbances in CD8^+^ T cells, undermining durable killing capacity and consolidating exhaustion states (153, 155). Notably, a recent review reported that, within chronic inflammation–associated HCC microenvironments, TAMs can erode CD8^+^ T-cell effector function and drive exhaustion through mechanisms such as reduced arginine availability and hypoxia-related pressures (143); these pathways are consistent with the metabolic stress and hypoxic features commonly observed in MASLD-associated HCC. Collectively, improving responses to immunotherapy is unlikely to rely on releasing a single checkpoint alone; rather, durable restoration of CD8^+^ T-cell function will likely require concurrent disruption of TAM-mediated inhibitory interactions and the metabolic/hypoxic stresses that sustain exhaustion, thereby reducing the risk of therapeutic refractoriness (139, 143).

## 4 Targeted therapy for liver macrophages: from CLD (fatty liver/liver fibrosis) to liver cancer

Given the pivotal role of macrophages in driving the continuum from chronic liver inflammation to malignancy, therapeutic strategies targeting their recruitment, polarization, and metabolic functions have emerged as promising interventions. We provide a comprehensive summary of these macrophage-centric mechanisms and corresponding therapeutic targets for MASLD, hepatic fibrosis, and hepatocellular carcinoma in Table 2. The following sections detail these specific strategies across the different stages of liver disease progression.

**TABLE 2:** Targeting macrophage-centric mechanisms for the treatment of MASLD, hepatic fibrosis, and hepatocellular carcinoma.

Stage of the disease	Treatment strategies	Therapeutic target	Mechanism of action	References
Metabolic dysfunction-associated steatotic liver disease/Simple fatty liver disease and non-alcoholic steatohepatitis	Drugs directly target macrophage receptors	CD163; GR-MD-02; CCR2	Anti-CD163-dexamethasone: Targets CD163 to deliver dexamethasone. GR-MD-02: Blocks galectin-3 on macrophages. Cenicriviroc: Inhibits CCR2 to reduce monocyte recruitment to the liver and acts on HSCs to affect fibrosis.	(10)
	Drug targeting of multiple pathogenic signaling pathways	FXR agonists; GLP1RAs	FXR agonists: Reduce pro-inflammatory cytokines and promote anti-inflammatory macrophage polarization. GLP1RAs: Decrease macrophage infiltration and promote anti-inflammatory macrophage polarization via GLP-1 receptors.	
	Regulates macrophage polarization and metabolism RNA methylation modifications	Metabolic reprograming; Macrophage polarization	The shift from aerobic glycolysis to oxidative phosphorylation promotes macrophage polarization from M1 to M2; m6A modification and its related enzymes regulate macrophage polarization, with METTL3, METTL14, FTO, YTHDF1, and YTHDF2 upregulated and WTAP and YTHDC2 downregulated.	(163, 164)
Liver fibrosis/Cirrhosis	Targeting macrophage immune metabolism	ACC inhibitors; PPAR; FXR	Prevent macrophage activation and infiltration; Promoting macrophage differentiation toward M2; Increasing cholesterol transport in macrophages	(22)
	Targeting macrophage-related signaling pathways	Antibiotics; IL-1β antagonists;CCR2/5 antagonists; Gal-3 antagonists	Removes intestinal bacteria and inhibits macrophage activity; Inhibit the activation of inflammasomes; Inhibition of monocyte recruitment; Inhibition of inflammatory macrophage function; Proreparative macrophages reverse liver fibrosis	
	Targeting autologous macrophages	CD45^+^CD14^+^25F9^hi^ cells	Proreparative macrophages reverse liver fibrosis	
Hepatocarcinoma	Inhibition of monocytes recruitment	CCR2 antagonist; CCL2; Glypican-3;	Inhibiting monocyte recruitment blocks the source of pro-tumor macrophages and thus suppresses hepatocellular carcinoma progression	(190, 191, 221, 227, 228)
	Eliminating TAMs	TAMs; Glypican-3	Eliminating TAMs relieves immunosuppression, blocks tumor angiogenesis and thus suppresses hepatocellular carcinoma progression	
	Reprograming TAM phenotype	CSF-1 receptor; CD163	Reprograming TAM phenotype converts them from pro-tumor to anti-tumor type, thereby suppressing hepatocellular carcinoma progression.	
	Targeting phagocytic checkpoints	CD47	Promote phagocytosis of macrophages	
	Cell therapy and delivery platforms	TAMs	Macrophage-targeted cell therapies and nanodelivery platforms enable tumor control and TAM functional modulation via engineering or precise molecular delivery.	

CCR, CC chemokine receptor; HSCs, Hepatic Stellate Cells; FXR, Farnesoid X receptor; GLP1RAs, Glucagon-like peptide-1 receptor agonists; Gal-3, galectin-3; ACC, acetyl-CoA carboxylase; PPAR, Peroxisome Proliferator-Activated Receptor; IL, Interleukin; CCL, C-C Motif Chemokine Ligand; TAMs, Tumor-Associated Macrophages; CSF, Colony-Stimulating Factor.

### 4.1 Treatment of simple fatty liver and non-alcoholic steatohepatitis

In the treatment of simple fatty liver and MASH, intervention strategies targeting macrophages mainly include three directions: direct targeting, indirect regulation, and metabolic reprograming. The direct targeting strategy regulates macrophage function through specific targets, such as anti-CD163-dexamethasone conjugated drugs that can precisely deliver drugs to macrophages, alleviating liver inflammation and fibrosis (156, 157); CCR2/CCR5 antagonist cenicriviroc delays disease progression by inhibiting monocyte recruitment to the liver (158). The indirect regulation strategy affects macrophages by modulating key signaling pathways, such as Farnesoid X receptor (FXR) agonist obeticholic acid, which can induce macrophages to polarize toward an anti-inflammatory phenotype (159, 160); Peroxisome Proliferator-Activated Receptor (PPAR) family agonists regulate macrophage inflammatory responses and numbers through different mechanisms (161, 162). The metabolic reprograming strategy focuses on the metabolic restructuring of macrophages, including triptolide promoting macrophage polarization toward M2 type by regulating Pyruvate Kinase M2 (163), and m6A methyltransferase METTL3 regulating macrophage metabolic reprograming and TGF-β1 secretion through epigenetic mechanisms, thereby influencing the progression of MASH (164). These multi-layered therapeutic strategies provide new directions for the treatment of fatty liver and MASH.

### 4.2 New strategies for targeting macrophages in the treatment of liver fibrosis/cirrhosis

Liver fibrosis and cirrhosis, as common terminal pathological outcomes of CLD, are characterized by excessive deposition of ECM. Macrophages play a dual role in this process: they are both drivers of fibrosis progression and executors of fibrosis regression, making them important therapeutic targets.

#### 4.2.1 Dynamic regulation of macrophages in the fibrosis process

In the early stages of chronic liver injury, liver-resident KCs and Ly6C^hi^ monocytes recruited via the CCL2-CCR2 axis polarize to the M1 phenotype (165), activating HSCs by secreting factors such as TGF-β and PDGF, which promotes collagen production (166). During this stage, macrophages highly express TIMP-1, inhibiting the activity of MMP and driving the progression of fibrosis (167). However, after the removal of injury stimuli, macrophages can convert to an M2-like phenotype (94), upregulating the expression of MMP-9 and MMP-12 while downregulating TIMP-1, facilitating ECM degradation and apoptosis of myofibroblasts, thereby achieving fibrosis reversal (168, 169).

#### 4.2.2 New advances in cell therapy

Based on the repair function of macrophages, autologous macrophage infusion therapy has entered the clinical validation stage. The Multicenter Autologous Monocyte-Derived Macrophage Therapy for Cirrhosis (MATCH) phase II clinical trial showed that although there was no significant improvement in Model for End-Stage Liver Disease (MELD) scores after the infusion of autologous MoMFs in patients with compensated liver cirrhosis, it demonstrated good safety and a lower incidence of liver-related serious adverse events. Patients in the treatment group exhibited elevated serum IL-15 levels and decreased IL-1β levels, presenting an anti-inflammatory cytokine profile (170), which lays the clinical foundation for macrophages as “living drugs” in the treatment of advanced liver disease.

#### 4.2.3 Multidimensional strategies for drug intervention

Current drug development mainly focuses on three directions: blocking macrophage recruitment, eliminating pathological macrophages, and reprograming macrophage function. The CCR2/CCR5 dual antagonist Cenicriviroc demonstrated clear anti-fibrotic efficacy in the Century Trial of Cenicriviroc in Non-alcoholic Steatohepatitis with Fibrosis (CENTAUR) trial, doubling the fibrosis improvement rate in MASH patients by blocking monocyte liver infiltration (171). Although CSF1R inhibitors can effectively deplete pathological macrophages (172), the safety issues arising from their systemic effects still need to be addressed. The most promising strategy is to regulate macrophage phenotype through metabolic reprograming, such as using Carnitine Palmitoyltransferase 1a inhibitors or PPAR inhibitors to intervene in FAO metabolism (173), which can reverse the pro-fibrotic M2 phenotype to the anti-fibrotic M1 phenotype, opening new avenues for the treatment of liver fibrosis.

### 4.3 Targeting TAMs in combined treatment strategies for liver cancer

#### 4.3.1 Tumor-promoting mechanisms of TAMs and therapeutic targets

TAMs drive the malignant progression of liver cancer through multiple mechanisms. First, as major architects of the immunosuppressive microenvironment, they construct an inhibitory immune barrier by secreting inhibitory cytokines such as IL-10 and TGF-β, and recruiting regulatory T cells (174). Second, as key regulators of angiogenesis, they promote pathological neovascularization by secreting factors like VEGF and MMP-9 (175–177). Third, by secreting IL-6, they activate the JAK/STAT3 signaling pathway to promote tumor cell proliferation (104, 106), while also facilitating invasion and metastasis by secreting MMP that degrade the ECM (178). Additionally, TAMs mediate treatment resistance to sorafenib, anti-VEGF drugs, and immune checkpoint inhibitors by activating alternative signaling pathways (141, 142). These mechanisms collectively establish the important position of TAMs as key therapeutic targets.

#### 4.3.2 Therapeutic strategies targeting TAMs

Based on recent advances in understanding the tumor-promoting roles and cellular heterogeneity of TAMs in HCC, particularly at the single-cell transcriptomic level (179, 180), multiple multidimensional and synergistic therapeutic strategies have been proposed and are being actively explored. These approaches aim to reduce TAM abundance, reprogram their functional phenotypes, restore their phagocytic capacity, or exploit TAMs as therapeutic effectors, as detailed below.

##### 4.3.2.1 Depleting TAMs and blocking recruitment

This strategy aims to reduce TAM accumulation in the TME by limiting monocyte influx and disrupting TAM survival signals. First, blockade of the CCL2/CCR2 axis can reduce the recruitment of inflammatory monocytes and TAM infiltration, thereby suppressing tumor growth and reshaping anti-tumor immunity in HCC models (181). Second, targeting the CSF1–CSF1R pathway can interfere with TAM maintenance and function; CSF1R blockade with PLX3397 in HCC models delayed tumor growth primarily by altering TAM polarization rather than simply depleting macrophages (182). Given the essential roles of macrophages in tissue homeostasis, systemic toxicities and on-target immune perturbations should be carefully monitored when applying CSF1R-directed agents (183).

##### 4.3.2.2 Reprograming TAM phenotype

An emerging approach focuses on re-educating TAMs from an immunosuppressive, tumor-promoting M2-like state toward an inflammatory, tumoricidal M1-like phenotype. Mechanistically, PI3Kγ acts as a key molecular switch in myeloid cells that regulates the balance between immune stimulation and immune suppression; inhibiting PI3Kγ signaling can reprogram macrophage transcriptional programs and enhance anti-tumor immunity (184). In addition, epigenetic modulation has been explored as a macrophage-reprograming strategy; DNMT inhibition has been reported to influence macrophage functional states in relevant inflammatory contexts (185). Collectively, these interventions aim to restore antigen presentation, promote pro-inflammatory cytokine production, and improve responsiveness to other immunotherapies.

##### 4.3.2.3 Targeting phagocytic checkpoints

Targeting macrophage-specific “don’t-eat-me” signals has become a major direction to restore TAM phagocytosis. CD47 blockade has been shown to promote anti-tumor immunity in liver cancer models by enhancing innate and downstream immune activation (including a CD103^+^ dendritic cell–NK cell axis) (186). Clinically, evorpacept (ALX148), a high-affinity CD47 blocker with an inactive Fc region, has been evaluated in a first-in-human phase 1 study (alone and in combinations), supporting the translational potential of CD47/SIRPα-pathway targeting (187). Beyond CD47, additional innate checkpoints are being explored: CD24–Siglec-10 signaling functions as a macrophage inhibitory axis and represents another targetable phagocytic checkpoint (188).

##### 4.3.2.4 Cell therapy and delivery platforms

Cell-based strategies targeting macrophages are advancing rapidly. CAR-macrophage therapy (e.g., CT-0508) has been evaluated in a phase 1 trial in HER2-overexpressing advanced solid tumors, highlighting the feasibility of engineered macrophage approaches for tumor control and microenvironment remodeling (189). Meanwhile, nanotechnology-based delivery platforms are increasingly used to target TAMs with improved precision. For example, biomimetic nanoparticles incorporating manganese dioxide and macrophage-membrane-based designs have been developed for TAM targeting and repolarization (190). In addition, mannose-decorated nanotherapeutics delivering TLR7/8 agonists have been reported to modulate TAM/TME immunity and enhance anti-tumor responses, supporting the rationale of ligand-guided TAM targeting (191).

Finally, combination regimens are emphasized: TAM reprograming approaches may synergize with immune checkpoint blockade by simultaneously reducing immunosuppression and improving adaptive immune activation, supporting the concept of multi-pronged TAM-centered immunotherapy (183).

## 5 Conclusion and outlook

Hepatic macrophages operate across the MASLD–MASH–fibrosis–HCC continuum and constitute a central hub linking metabolic dysregulation, chronic inflammation, tissue remodeling, and tumor immune escape. Rather than a single class of inflammatory effectors, they form a heterogeneous and highly plastic cellular network: in early disease, macrophages help maintain hepatic homeostasis through lipid and debris clearance, containment of inflammatory spread, and promotion of tissue repair; under sustained pressures—such as lipotoxicity, low-grade chronic inflammation, and microbiome perturbation—macrophage programs can progressively drift toward pro-inflammatory, pro-fibrotic, and ultimately immunosuppressive states, thereby facilitating fibrotic accumulation and establishing a permissive microenvironment for tumor initiation and progression.

Importantly, explaining clinical heterogeneity and enabling precision intervention require moving beyond a framework centered solely on canonical pathways and incorporating several rapidly emerging dimensions. First, sexual dimorphism can systematically reshape macrophage states and influence disease trajectories: estrogens generally promote anti-inflammatory and repair-associated programs via ERα signaling, whereas androgens may reinforce pro-inflammatory features through NF-κB-related pathways. This may partially account for the higher risk of progression in men and postmenopausal women, supporting sex-aware stratification and hormone-axis–informed therapeutic design (192–195). Second, trained immunity provides a mechanistic basis for chronic, relapse-prone inflammation. Metabolic stress and microbe-associated stimuli can induce durable epigenetic marks (e.g., H3K4me3 and H3K27ac) accompanied by metabolic rewiring (e.g., enhanced glycolysis and succinate accumulation), locking macrophages into a long-lasting hyper-responsive state; upon secondary challenges, amplified inflammatory outputs can sustain chronic injury, promote fibrotic remodeling, and facilitate oncogenic transformation (196, 197). Third, gut–liver immunometabolic crosstalk extends far beyond LPS: microbiota-derived metabolites can directly “tune” macrophage polarization and inflammatory thresholds. For example, Trimethylamine N-oxide (TMAO) has been associated with an M1-leaning program and increased pro-inflammatory mediators (198, 199). Bile-acid profiles can exert bidirectional control through the FXR/TGR5 axis, and dysbiosis-driven bile-acid dysregulation may disrupt this balance (200–204). Tryptophan metabolites can also shape macrophage programs via AhR-related pathways, with indole derivatives generally supporting anti-inflammatory responses, whereas kynurenine-linked signaling has been associated with pro-fibrotic activation (205, 206). Collectively, these dimensions underscore that macrophages are active integrators within metabolic–immune–microbial networks rather than passive responders, and that their state trajectories can determine both the direction and tempo of transitions from metabolic inflammation to structural damage and tumor niche formation.

Along the disease chain, macrophage functions are stage-dependent. In early MASLD, macrophages primarily preserve hepatic homeostasis by regulating lipid metabolism and inflammatory thresholds (207). As disease progresses to MASH, KC activation and monocyte recruitment constitute key events that amplify inflammation, while the formation of CLS and expansion of inflammation-associated macrophage subsets provide a structural basis for self-sustaining chronic inflammation (76). During fibrosis, macrophages can exert time-window–dependent bidirectional effects on fibrogenesis and regression, with net outcomes shaped by cellular origin, functional state, and spatial distribution. In HCC, TAMs sculpt an immune-evasive niche via immunosuppression, angiogenesis, and metabolic reprograming, thereby promoting tumor progression and therapeutic resistance. Across stages, a unifying theme is that “time–space–function” remodeling of macrophage states represents a key variable enabling cross-stage transitions.

From a translational perspective, macrophage-targeted interventions are shifting from single-axis anti-inflammatory “depolarization” toward multidimensional, stage-tailored precision regulation. CCR2/CCR5 antagonists, CSF1R inhibitors (208), antibodies targeting TREM2 or MARCO (209), and nanoparticle-enabled siRNA/exosome strategies (210) have shown promise in clinical or preclinical settings. Meanwhile, interventions aligned with the emerging dimensions are coming into view, including selective ERα agonists for sex-informed modulation, epigenetic regulators (e.g., HDAC inhibitors) to reset trained-immunity–linked hyper-responsiveness (211, 212), and indirect reshaping of gut–liver immunometabolic dialogue through inhibition of TMAO synthesis or modulation of bile-acid receptors (213). Nevertheless, functional overlap, continuum-like phenotypes, and dynamic interconversion among macrophage populations render “precisely targeting pathogenic programs while preserving repair” a central bottleneck. The chemokine network and parallel inflammatory cues are highly redundant, such that single-axis recruitment blockade (e.g., CCR2/CCR5) can be bypassed by compensatory pathways, limiting both efficacy and durability (214, 215). Moreover, pathogenic subsets partially overlap with homeostatic and repair-associated macrophages in markers and functional modules, and can rapidly transition across spatial niches, complicating selective targeting (216, 217). In addition, pathway blockade may trigger compensatory rewiring at network level (e.g., alternative recruitment, adaptive myeloid remodeling, and reconfigured immune cross-talk), further undermining durability and reinforcing the need for rational combinations and stage-/sequence-adapted strategies (217). Accordingly, future progress will likely rely on multi-target synergy and temporally sequenced regimens, supported by quantifiable immunometabolic biomarkers for stratification and longitudinal monitoring to achieve a more robust balance between efficacy and safety.

Future work may prioritize four directions: (1) constructing spatiotemporal atlases of macrophage subsets using single-cell and spatial multi-omics to resolve fate trajectories and key regulatory nodes (218, 219); (2) elucidating macrophage migration and signal integration in inter-organ crosstalk, particularly within systemic inflammatory loops involving the gut–liver axis, adipose–liver, and bone marrow–liver connections; (3) developing modular, synergistic intervention systems that integrate metabolic regulation, drugs/antibodies, gene delivery, and immunotherapy to enable stage-tailored disruption of pathogenic programs; and (4) establishing immunometabolic biomarker systems for response prediction and relapse warning, centered on macrophage subset proportions, metabolic states, and pathway activities.

In summary, hepatic macrophages constitute an “immunometabolic regulatory axis” spanning the MASLD–HCC continuum. Their state remodeling is both a major driver of disease progression and a therapeutically exploitable vulnerability. With the convergence of high-resolution multi-omics and emerging immunotherapeutic strategies, controllable reprograming of macrophage states may become feasible, advancing liver-disease prevention, intervention, and precision therapy into a more verifiable, stratified, and iterative new phase.
